# Case Report: Challenges of Non-Invasive Prenatal Testing (NIPT): A Case Report of Confined Placental Mosaicism and Clinical Considerations

**DOI:** 10.3389/fgene.2022.881284

**Published:** 2022-05-12

**Authors:** Giulia Bonanni, Valentina Trevisan, Marcella Zollino, Marco De Santis, Federica Romanzi, Antonio Lanzone, Elisa Bevilacqua

**Affiliations:** ^1^ Unit of Obstetrics and Gynecology, Università Cattolica del Sacro Cuore, Rome, Italy; ^2^ Unit of Medical Genetics, Fondazione Policlinico Universitario A. Gemelli IRCCS, Rome, Italy; ^3^ Section of Genomic Medicine, Department of Life Sciences and Public Health, Università Cattolica del Sacro Cuore, Rome, Italy; ^4^ Department of Women and Child Health, Women Health Area, Fondazione Policlinico Universitario Agostino Gemelli IRCCS, Rome, Italy

**Keywords:** NIPT, cfDNA, confined placental mosaicism, prenatal diagnosis, aneuploidies

## Abstract

Since the introduction of cell-free (cf) DNA analysis, Non-Invasive Prenatal Testing (NIPT) underwent a deep revolution. Pregnancies at high risk for common fetal aneuploidies can now be easily identified through the analysis of chromosome-derived components found in maternal circulation, with the highest sensitivity and specificity currently available. Consequently, the last decade has witnessed a widespread growth in cfDNA-based NIPT use, enough to be often considered an alternative method to other screening modalities. Nevertheless, the use of NIPT in clinical practice is still not devoid of discordant results. Hereby, we report a case of confined placental mosaicism (CPM) in which a NIPT false-positive result for trisomy 13 required not only amniocentesis but also cordocentesis, to rule out the fetal aneuploidy, with the additional support of molecular cytogenetics on placental DNA at delivery. Relevant aspects allowing for precision genetic diagnosis and counselling, including the number of analysed metaphases on the different fetal cells compartments and a repeated multidisciplinary evaluation, are discussed.

## Introduction

Cell-free (cf) DNA-based Non-Invasive Prenatal Testing (NIPT) is widely considered to be the most sensitive and specific screening option for trisomy 21, 18, and 13. However, some concerns regarding its clinical role in routine obstetric care persist. These include, *inter alia*, the reliability of Positive Predictive Value (PPV) estimates. According to the most recent metanalyses (Gil et al., 2015; Taylor-Philips et al., 2016; Iwarsson et al., 2017; Mackie et al., 2017), the combined False-Positive Rate (FPR) in successful tests is 0.15%. In this sense, most studies on NIPT performance still suffer from a high risk of bias, in particular, the reported FPRs are likely to be underestimated.

As it is well known, circulating cfDNA derives from both the mother and the fetal-placental unit. Consequently, the main sources of unreliability of NIPT are Confined Placental Mosaicism (CPM), maternal copy number variants, vanishing twin, and maternal cancer (Grati et al., 2014).

Despite these shortcomings, obstetric care providers are increasingly prone to prescribe cfDNA analysis as an alternative or stand-alone screening method compared to ultrasound examinations. Moreover, there is still some controversy concerning the standard protocols that would best investigate fetal anomalies during the first trimester.

With this in mind, we report a case of CPM in which a NIPT false-positive result for trisomy 13 required two further invasive diagnostic tests–an amniocentesis and a cordocentesis - to rule out the fetal aneuploidy. Molecular cytogenetics performed on placental DNA at the delivery could add relevant data for the unequivocal diagnosis of CPM.

### Case Presentation

**FIGURE 1:**
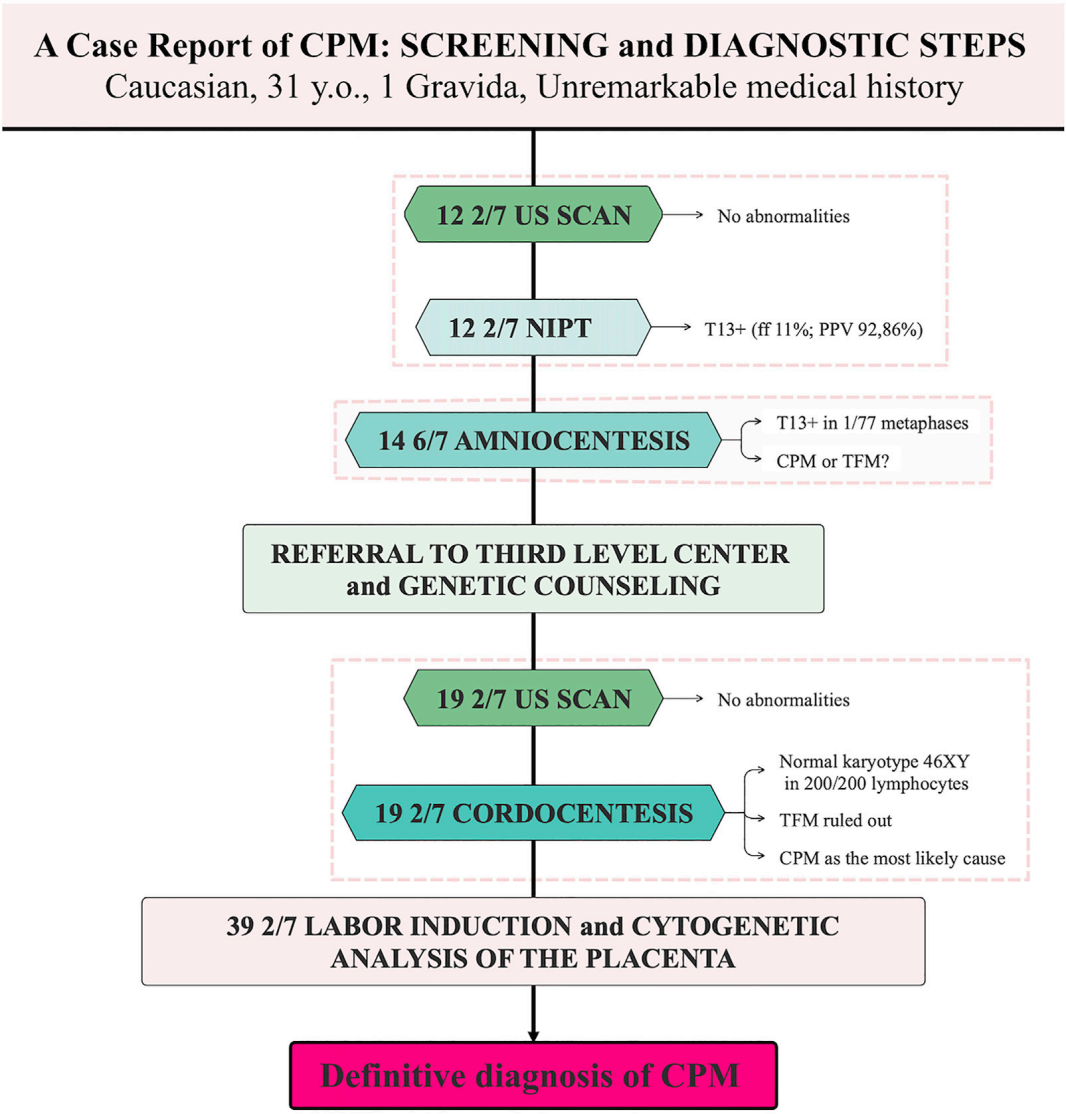
Screening and diagnostic steps in a case of Confined Placental Mosaicism. CPM, Confined Placental Mosaicism; TFM, True Fetal Mosaicism; ff, Fetal Fraction; PPV, Positive Predictive Value.

A 31-year-old, gravida 1, para 0, Caucasian woman was referred to our hospital (Agostino Gemelli University Policlinic, Rome, RM, Italy) at 19 2/7 weeks of gestation for evaluation of suspected trisomy 13. Her previous medical and obstetric history had been unremarkable. Screening and diagnostic steps are presented in Figure 1. The first-trimester ultrasound findings were normal. At 12 2/7 weeks of gestation, she underwent the PrenatalSAFE® 5 Test (Eurofins Genoma Group Srl, Rome, Italy) through ILLUMINA VeriSeq NIPT sequencing systems, which revealed a suspected aneuploidy in chromosome 13 with a Fetal Fraction (FF) of 11% and a PPV of 92,86%. At 14 6/7 weeks of gestation, she underwent amniocentesis to confirm the positive NIPT result. By analysing 77 metaphases, we found that all but one had a normal male chromosome constitution, 46 XY. The unique cell with trisomy 13 we observed was first consistent with CPM, or, alternatively, with a very low mosaicism for trisomy 13 in the fetus.

A detailed fetal ultrasound was carried out at 19 2/7 weeks of gestation with normal results.

At 19 2/7 weeks of gestation, upon genetic counselling, the couple also decided to undergo cordocentesis to rule out a True Fetal Mosaicism (TFM) for trisomy 13. The analysis of 200 fetal lymphocytes confirmed a normal male karyotype 46, XY in the totality of cells. Accordingly, CPM was considered to explain the previous results.

Subsequent ultrasound scans proved to be within normal limits.

Labor induction was performed at 39 2/7 weeks of gestation for reduction of the fetal growth trend (from 64th to 23rd percentile), that was considered to reflect the final functional dysfunction of the trisomic placenta. A male neonate with a birth weight of 3430 g and Apgar scores of 9–10 at 1–5 min, respectively, was born by vaginal delivery. Physiological newborn jaundice was present, and the infant did not present any phenotypic anomaly. Molecular cytogenetic examination (Array-CGH) of the placenta, revealed a complete trisomy of chromosome 13 in about 20% of the analysed genome, allowing to definitively establish the diagnosis of CPM. During a final genetic counselling, no risk for phenotypic abnormalities was given to the newborn, and amniocentesis for fetal chromosomal examination was suggested in subsequent pregnancies of the parents.

## Discussion

In a seminal paper, Lo and Wainscoat (Lo et al., 1997) described for the first time the presence of fetal DNA in maternal plasma. Since then, several studies (Sekizawa et al., 2000; Bianchi, 2004; Tjoa et al., 2006) have been carried out to investigate cfDNA mechanisms of release during pregnancy, demonstrating both the maternal and the fetal-placental unit origin. Despite the high sensitivity and specificity of currently available PCR and MPS analytical techniques for the study of cfDNA (Bianchi et al., 2012), the primary trophoblastic origin of the latter is a known driver of the relatively large number of false positive and false negative results (Grati et al., 2014). The upshot of this is the designation of NIPT as a screening - and not a diagnostic - test. In this context, the present case highlights the shortcomings of NIPT when used as an alternative to the first trimester ultrasound scan for the screening of the most common aneuploidies. We aim at evaluating some critical aspects that should be considered in protocol decision-making practices and which could best investigate fetal anomalies during the first trimester.

First, it is interesting to note that Fetal Blood Sampling (FBS) through cordocentesis can sometimes be diriment to rule out a fetal aneuploidy after a NIPT positive result. In our case, normal ultrasound findings against the background of a positive result for trisomy 13 at NIPT led initially to consider amniocentesis the best diagnostic tool to avoid erroneous results due to CPM. Despite this, cordocentesis was then deemed necessary to exclude a fetal mosaicism. This means not only exposure to all the potential risks of FBS - including bleeding from the puncture site, fetal bradycardia, pregnancy loss and vertical transmission of maternal infection (Berry et al., 2013) - but also a 47-days delay in the final response, resulting in substantial psychological stress over a long period. Such an emotional strain should be avoided since it could lead, in extremis, to an improper decision to have a first-trimester abortion for the sake of the mother’s health.

This emphasizes, above all, how important both pre- and post-test counselling are, allowing patients to understand the difference between a screening and a diagnostic test. In this sense, we believe that the best prenatal practice encompasses the interpretation of both positive and negative NIPT results in view of other screening modalities’ findings (Salomon et al., 2017). Conversely, most laboratories report the average risk in the screen-positive patient as a PPV, disregarding the prior-test risk based on age, ultrasound, prior history and screen-positive serum test. Abnormal findings at NIPT, contrasting with normal fetus development at ultrasound scan, could disclose other biological causes (Hartwig et al., 2017), such as maternal Copy Number Variations (CNVs) and Confined Placental Mosaicism (CPM) (Mardy & Wapner, 2016). In this context, even if the performance of NIPT is higher, the first trimester ultrasound scan has been proved to potentially change clinical management in almost one in 10 women if performed prior to cfDNA screening (Brown et al., 2020). This is especially the case of trisomies 18 and 13, for which a detailed ultrasound examination can detect characteristic defects.

The present case also maintains the need of a careful perinatal management when CPM is suspected. After ruling out recognized risk factors such as constitutional chromosomal abnormalities, the rate of infants with Intrauterine Growth Restriction (IUGR) associated with CPM has been estimated to be 10 times higher than in the appropriately grown controls infants (Wilkins-Haug et al., 2006). Notwithstanding this, a recent retrospective cohort study did not confirm any significant association between CPM and adverse pregnancy outcomes except for CPM for trisomy 16 (Grati et al., 2020).

In conclusion, this case provides significant clinical considerations on using NIPT in daily practice. In particular, it presents the major pitfalls of interpreting screening tests’ findings in the absence of a mutual work of integration. In recent years, we have witnessed an uncontrolled spread of cf-DNA analysis for which the economic interest of the industry has certainly contributed. In this context, a responsible integration of similar technological innovations should always be sought in clinical experience. Our clinical experience confirms that, given the trophoblastic origin of cf-DNA, NIPT cannot but be a screening test. The real benefit of cfDNA analysis lies, therefore, in its complementary use with ultrasound scan, which helps to shed light on the most likely risk of fetal aneuploidy. We suggest that, in case of cfDNA testing positive for T21, T18, and T13 during first trimester screening, in the absence of anomalies detected during the ultrasound examination, an invasive procedure by Chorionic Villus Sampling (CVS) could be recommended only for T21, since in such a case the risk of confined placental mosaicism is about 1–2%, which is comparable to the risk of mosaicism in the general population. Conversely, for T18 or T13, the best management would be to offer an amniocentesis because the risk of confined placental mosaicism is high: 3–4% for T18 and 22% for 13 (Grati et al., 2014; Grati et al., 2015; Malvestiti et al., 2015). However, based on our observation, cordocentesis can be also required to definitively rule out the fetal aneuploidy. Analysis of a larger number of metaphases from fetal blood cells, with respect to amniocytes, is recommended in these cases.

Finally, we strongly emphasize the importance of an adequate education of all obstetrical providers in order to maximize the benefit brought by cfDNA analyses. Moreover, we notice that further research is needed to examine the extent to which maternal risk factors (e.g., age, obesity, hypertension, diabetes) influence the incidence of IUGR associated to CPM.

## Data Availability

The datasets for this article are not publicly available due to concerns regarding participant/patient anonymity. Requests to access the datasets should be directed to the corresponding author.
